# Intra-articular injection of a nutritive mixture solution protects articular cartilage from osteoarthritic progression induced by anterior cruciate ligament transection in mature rabbits: a randomized controlled trial

**DOI:** 10.1186/ar2114

**Published:** 2007-01-26

**Authors:** Yoo-Sin Park, Si-Woong Lim, Il-Hoon Lee, Tae-Jin Lee, Jong-Sung Kim, Jin Soo Han

**Affiliations:** 1 Institute of Biomedical Science, College of Medicine 1F, Hanyang University, Haengdang-dong 17, Seongdong-gu, Seoul, 133-791, South Korea; 2 Department of Physical Medicine and Rehabilitation, School of Medicine, Inje University, Gaekeum-dong 633-165, Pusanjin-gu, Pusan, 614-735, South Korea; 3 Chamsarang PM&R Clinic, Chonho-dong 455, Gangdong-gu, Seoul, 134-020, South Korea; 4 Kwangmyung PM&R Clinic, Kwangmyung-dong 340-5, Kwangmyung, Gyunggi-do, 423-016, South Korea; 5 Department of Pathology, School of Medicine, Chungang University, Heukseok-dong, Dongjak-gu, Seoul, 155-756, South Korea; 6 Laboratory Animal Research Center, Samsung Biomedical Research Institute, Samsung Medical Center, Ilwon-dong 50, Gangnam-gu, Seoul, 135-710, South Korea; 7 Department of Laboratory Animal Medicine, College of Veterinary Medicine, Konkuk University, Hwayang-dong 1, Gwangjin-gu, Seoul, 143-701, South Korea

## Abstract

Osteoarthritis (OA) is a degenerative disease that disrupts the collagenous matrix of articular cartilage and is difficult to cure because articular cartilage is a nonvascular tissue. Treatment of OA has targeted macromolecular substitutes for cartilage components, such as hyaluronic acid or genetically engineered materials. However, the goal of the present study was to examine whether intra-articular injection of the elementary nutrients restores the matrix of arthritic knee joints in mature animals. A nutritive mixture solution (NMS) was composed of elementary nutrients such as glucose or dextrose, amino acids and ascorbic acid. It was administered five times (at weeks 6, 8, 10, 13 and 16) into the unilateral anterior cruciate ligament transected knee joints of mature New Zealand White rabbits, and the effect of NMS injection was compared with that of normal saline. OA progression was histopathologically evaluated by haematoxylin and eosin staining, by the Mankin grading method and by scanning electron microscopy at week 19. NMS injection decreased progressive erosion of articular cartilage overall compared with injection of normal saline (*P *< 0.01), and nms joints exhibited no differences relative to normal cartilage that had not undergone transection of the anterior cruciate ligament, as assessed using the mankin grading method. Haematoxylin and eosin staining and scanning electron microscopy findings also indicated that nms injection, in constrast to normal saline injection, restored the cartilage matrix, which is known to be composed of a collagen and proteoglycan network. thus, nms injection is a potent treatment that significantly retards oa progression, which in turn prevents progressive destruction of joints and functional loss in mature animals.

## Introduction

Osteoarthritis (OA) is induced by complex mechanisms such as progressive erosion of articular cartilage, proteoglycan (PG) degradation and disruption of the collagen network, all of which lead to progressive destruction of joints and functional loss [1]. Until recently the only therapies available to patients with OA were short-term relief agents [1-3], oral nutrient supplements [4-6], proliferative or regenerative therapies [7-10] and total surgical replacement of articular cartilage [11]. In Korea, intra-articular injection is becoming increasingly popular because of its convenience and rapid effects. Agents that are commonly administered by injection include analgesics, nonsteroidal anti-inflammatory drugs (NSAIDs), steroids, hyaluronic acid and glucose [12]. Analgesics, NSAIDs and steroids have anti-inflammatory effects. However, analgesics and NSAIDs provide only temporary pain relief [3,13], and steroids are of limited use because of the resulting symptomatic 'dry' knees [12,14]. Hyaluronic acid improves only molecular-weight-related short-term viscoelasticity of the joint synovial fluid [15,16]. Injection of glucose or dextrose is used to manage chronic musculoskeletal pain, soft tissue injuries, and ligament and joint laxity [17]. Although the therapeutic effects of dextrose or glucose are stronger with increased concentration [18], severe pain caused by inflammatory reactions at the injection site can also occur as a result of increased concentration [19].

Most patients would prefer treatments that are inexpensive and have long-term efficacy, and are less painful, less invasive and more easily accessible, and with fewer side effects than with existing treatments [17]. Therefore, given the needs of OA patients and the limitations of existing OA treatments, we designed an intra-articular injection material that might confer greater therapeutic benefit in OA and fulfill patients' needs.

This material is a nutritive mixture solution (NMS), and it is formulated to supply nutrients to chondrocytes, which in turn synthesize collagen or proteoglycan (PG) to maintain the matrix network [20,21]. Collagen fibres, especially type II collagen and PG, hold water to give tensile and compressive stiffness, and cartilage integrity depends on a successful symbiotic relationship between chondrocytes and interstitial matrix [22,23]. NMS is composed of glucose or dextrose, several amino acids and ascorbic acid. Among the NMS components, glucose or dextrose plays a role in elevating levels of certain growth factors in ligaments after injury [24] and in serving as an energy substrate for chondrocytes and promoting matrix metabolism [20]. The amino acids that we selected are the substrates for fibril forming collagen or PG in articular cartilage. They include glycine, proline, hydroxyproline, glutamate, alanine, aspartate, serine, glutamine, arginine, lysine and methionine [25,26]. Cysteine is not a substrate of fibril forming collagen, but it protects cartilage from oxidative damage by acting as a thiol antioxidant [27]. The anti-OA roles that these amino acids play in articular cartilage have been examined in *in vitro *or *in vivo *studies [27-38]. Finally, ascorbic acid is required for synthesis of type II collagen and PG as a cofactor in articular cartilage [39,40].

In light of the physiological roles played by each of these elementary nutrients, we investigated the therapeutic effects of intra-articular injection of NMS on osteoarthritic knee joints, as compared with the effect of injection of normal saline (NS). The study was conducted using an experimental model in which OA develops as a result of anterior cruciate ligament transection (ACLT) in New Zealand White Rabbits with closed growth plates.

## Materials and methods

### Experimental materials

The NMS was composed of 20% dextrose or glucose solution, 20% amino acid solution and 5% ascorbic acid solution, and they were mixed at a ratio of 50:40:10 (Table 1). For injection, dextrose or glucose was finally diluted to a 10% solution, which has been shown to be the effective and most tolerable concentration and so the most acceptable to the patients [41]. The amino acids were selected based on their frequency of occurrence in fibril forming collagen, especially type II collagen; thus proline, hydroxyproline and glycine – the major amino acids required for the triple helical structure of collagen – were selected [25,42]. All of the remaining amino acids with a frequency of occurrence of about 10% or greater in the triple-helical structure of collagen were also selected. These minor amino acids were glutamate, alanine, aspartate, serine, glutamine, arginine, lysine and methionine. Cysteine and ascorbic acid were added as cofactors that promote the synthesis of type II collagen in articular cartilage [27,40]. Ascorbic solution should be made just before injection, because it is very unstable and highly reactive. The effect of injection of NS (0.9% sodium chloride solution) into osteoarthritic joints was also evaluated as a control intervention. Dextrose or glucose solution (20%) and sodium chloride solution (0.9%) were purchased from Daehan Pharmaceutical Co., Ltd (Seoul, South Korea) and all of the amino acids and ascorbic acid were purchased from Sigma Co., Ltd (St. Louis, MO, USA).

### Experimental animals

Twenty-four mature New Zealand White rabbits (female, age 9 ± 2 months, body weight 3.6 ± 0.2 kg) were examined in this study. Ten rabbits were from the Laboratory Animal Research Center, Samsung Biomedical Research Institute (Samsung Medical Center, Seoul, South Korea) and 14 were from the Laboratory Animal Research Center, ChemOn Institute (Yongin, Gyunggi-do, South Korea). The rabbits were housed individually and had free access to tap water and commercial rabbit diet. The animal experiments were performed in accordance with internationally accredited guidelines, and were approved by each laboratory's Institutional Animal Care and Use Committee.

### ACLT surgery for induction of osteoarthritis

Experimental OA in rabbits was induced by ACLT surgery in both groups. The rabbits were anaesthetized with intramuscular injection of ketamine (5 mg/kg) and butorphanol (0.1 mg/kg). After shaving and sterilizing the surgical site, ACLT was performed using a para-medial approach with the skin incision in the left knee medial para-patellar area. To achieve optimal visualization of the anterior cruciate ligament, the patellar bone was displaced laterally and the knee was placed in full flexion. The anterior stability was confirmed by an anterior drawer test [43]. The synovium and the incised skin were sutured, and sterile dressing was applied. Following the surgical procedure, gentamycin (5 mg/kg) was injected intramuscularly into each rabbit once daily for a week.

### Experimental protocol for treatment

**Figure 1:**
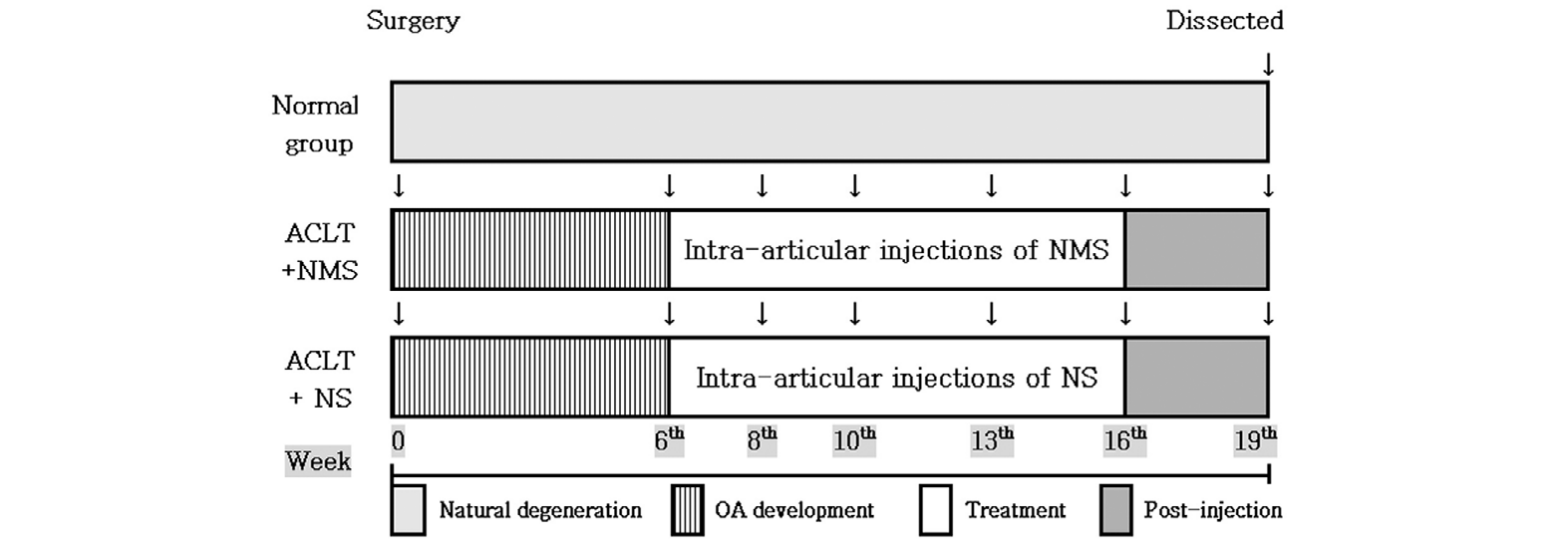
Schematic diagram of the experimental protocol. The Normal group includes right knee joints from the mature rabbits, which had not undergone any surgery or treatment (*n *= 7). In the ACLT+NMS group, joints had undergone anterior cruciate ligament transection (ACLT) surgery followed by injections of nutritive mixture solution (NMS; *n *= 7). In the ACLT+NS group, joints had undergone ATLC surgery followed by normal saline (NS) injections (*n *= 11). The intra-articular injection volume was 0.5 ml (NMS or NS), and injections were given for 13 consecutive weeks starting on the week 6 after surgery and with 2-week or 3-week intervals. All of the rabbits were dissected at week 19, and histopathological examinations (such as haematoxylin and eosin staining, Mankin grading method and scanning electron microscopy) were performed in all three groups.

A schematic diagram of the experimental protocol is presented in Figure 1. All the rabbits in both laboratories were evenly divided into two groups by weights a week after the ACLT procedure. Generally, articular cartilage exhibits degenerate changes approximately 3–8 weeks after ACLT in experimental rabbits [44,45]. Thus, 0.5 ml of each reagent was injected intra-articularly to the left knee with ACLT at week 6 after surgery. Four more injections were administered at 2-week or 3-week intervals at weeks 8, 10, 13 and 16 after surgery. One group was injected with NMS and the other was injected with NS. In both groups the intact right cartilage served as the normal articular cartilage (Normal) group, and did not undergo any treatment. We lost two rabbits because of infection in the NMS group, and the remaining rabbits were killed by infusion of potassium chloride 3 weeks after the last injection, at week 19 after ACLT. For the Normal group, samples were randomly taken from the untreated right knees of the rabbits in both groups: three from the NMS group and four from the NS group. Haematoxylin and eosin (H&E) staining was applied to tissue samples from each of the three groups. Scanning electron microscopy (SEM) images of the articular cartilage were also examined to confirm the histopathological findings in the three groups.

### Histopathological examinations: H&E staining

After the rabbits had been killed, the knee joints of the rabbits were dissected. The medial tibial plateaus and medial femoral condyles of the rabbits were fixed in 10% phosphate-buffered formalin (pH 7.4) with 1% cetylpyridinium (CPC) for 24 hours and decalcified with 20% EDTA. The decalcified specimens were embedded in paraffin and 1 μm thin sections were stained with H&E for light microscopic examination (×100) [46]. The severity of articular cartilage lesions was graded through double-blind observations, using the histological grading method proposed by Mankin and coworkers [47]. The Mankin grading method is a well known and proven method for the histological evaluation of OA cartilage. This method evaluates the severity of erosion and/or fissures of cartilage, disorganization or loss of chondrocytes, and pannus formation. Thus, the method adequately satisfies the criteria for measuring osteoarthritic changes in human and experimental animals [16,48]. Other parenchymal organs were also examined to investigate possible deleterious effects of the treatment material.

### Histopathological examinations: scanning electron microscopy

The extent of fibrillation and abrasion on the cartilage surface was observed in the photographs obtained by SEM (Joel 35CF; Tokyo, Japan; ×6,000). The microsections of cartilage of the medial tibial plateau, which is part of a weight-bearing joint, were washed with normal saline and pre-fixed in 2.5% glutaraldehyde-1/15 M phosphate buffer solution. After serial dehydration with ethanol, the ethanol was replaced with isoamyl acetate, and the samples were completely dried in a dryer. An ionic coater was used for gold deposition, and the coated samples were imaged by SEM [16].

### Statistical analysis

The histopathological evaluation gradings obtained using the Mankin grading method [47] were pooled for the normal group (*n *= 7), the NMS group (*n *= 7), and the NS group (*n *= 11). The mean values of the grades were compared among the three groups by one-way analysis of variance and the Tukey HSD test or Kruskal-Wallis test, depending on normality of data (*P *< 0.05).

## Results

### Histopathological results: H&E staining

**Figure 2:**
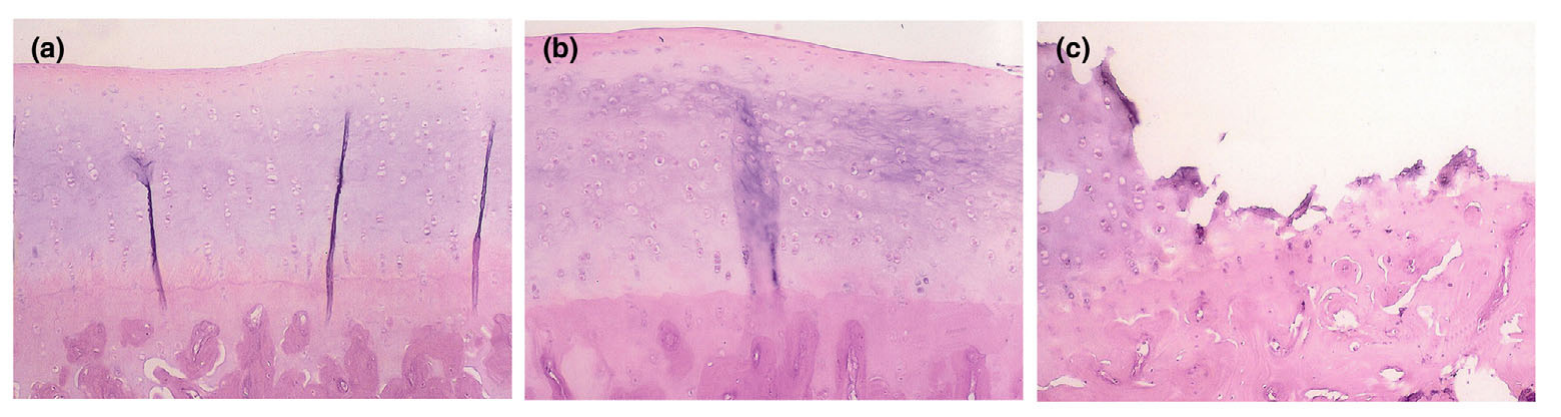
Representative photomicrographs of articular cartilage of the medial tibial plateau. These photomicrographs were taken at 19 weeks after anterior cruciate ligament transection surgery, the stain used is haematoxylin and eosin, and the magnificantion is ×100. **(a) **Normal group. **(b) **Nutritive mixture solution (NMS) group. **(c) **Normal saline (NS) group.

The cartilage surfaces of the weight-bearing parts, such as the medial tibial plateau and medial femoral condyle, were evaluated and graded for the extent of degradation. Table 2 presents the histopathological results of H&E staining using the Mankin grading method [47]. A set of photomicrographs by light microscopy is also presented in Figure 2, which shows the representative medial tibial plateaus in the three groups. The least changes were noted in the Normal group: slight surface irregularities, and slight to moderate hypercellularities in the transitional and radial zones (Table 2 and Figure 2a). In the NMS group the few changes noted were moderate surface irregularities, swelling of chondrocytes in the tangential zones, and moderate to severe hypercellularities in the transitional and radial zones (Figure 2b). However, in terms of the degenerative changes observed, there were no significant differences between the NMS group and the Normal group (Table 2). In addition, almost all of the histological changes in articular cartilage, especially the degenerative changes in the medial tibial plateau, were significantly less severe in the NMS group than in the NS group (*P *≤ 0.001). On the other hand, significant degenerative findings were noted in the NS group (Table 2 and Figure 2c), such as severe surface irregularities, a cleft in the radial zone, swelling or disappearance of chondrocytes in the tangential zone, moderate to severe cloning in the transitional and radial zones and slight pannus formation, as compared with the Normal and NMS groups (*P *≤ 0.01). In particular, in the NS group there was disappearance of surface layer cells, and loss of the cartilage matrix extended to the calcified zone (Figure 2c). These findings indicate that NMS injection reduced loss of the superficial layer and erosion of cartilage as compared with NS injection, and conferred protection effects against the OA-like degenerative changes in the articular cartilage. Other parenchymal organs taken from the treatment group did not exhibit any remarkable deleterious changes (data not shown).

### Histopathological results: scanning electron microscopy

**Figure 3:**
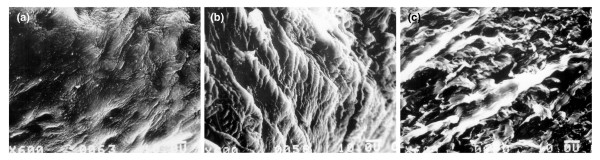
Scanning electron micrographs of articular cartilage surface of the medial tibial plateau. These micrographs were taken at 19 weeks after anterior cruciate ligament transection surgery and the magnification is ×6,000 (scale bar is 10 μm). **(a) **Normal group. **(b) **Nutritive mixture solution (NMS) group. **(c) **Normal saline (NS) group.

A set of samples evaluated by SEM shows the surface of the medial tibial plateau in the three groups (Figure 3). The Normal group exhibited the smoothest and the most intact superficial surface of the articular cartilage (Figure 3a). In the NMS group (Figure 3b), the medial tibial plateau was partially exposed where the cartilage matrix was missing. However, the medial tibial plateau of the NMS group was less damaged than that of the NS group, particularly in the full thickness and the superficial zone of the articular cartilage. The loss of cartilage matrix in the NMS group was obviously less severe than that in the NS group, and the configuration of the articular cartilage in the NMS group was much closer to that in the Normal group. As opposed to the NMS and Normal groups, in the NS group no cartilage matrix remained, and some of calcified layer of cartilage was exposed (Figure 3c).

## Discussion

Our experimental material, NMS, is a nutritive mixture solution that is designed to upregulate chondrocytes' regenerative potential to synthesize a collagen and PG network. The components of NMS are solutions of dextrose or glucose, amino acids and ascorbic acid. These elementary nutrients are substrates that can be delivered into the articular cartilage via the synovial route, which is a major nutrient transport pathway for ligaments and menisci of the articular joint [49]. Articular cartilage has extremely small pores (estimated at 50 Å) in the superficial zone, and so only low-molecular-weight compounds (<20 kDa) in synovial fluid may diffuse into the tissue [26]. All of the components of NMS can move freely through the tissue because they are not heavy molecular compounds. Moreover, articular chondrocytes have special transporter systems for glucose and ascorbic acid [50]. Glucose is delivered to the chondrocytes via synovial microcirculation and taken up by glucose uptake (GLUT) proteins. The intracellular glucose pool is used for glycolysis and extracellular matrix macromolecules [51]. The supply of glucose for anaerobic metabolism is essential to the survival and proliferation of chondrocytes and for the maintenance of matrix integrity. Therefore, impaired glucose uptake would compromise chondrocyte function, and potentially result in an imbalance in cartilage matrix synthesis and degradation, leading to OA [20]. Ascorbic acid is transported into chondrocytes by the sodium-dependent vitamin C transporter (SVCT2), and has been shown to upregulate the expression of type II collagen and aggrecan [52]. Ascorbic acid also plays an important role in chondrocyte proliferation and protection from oxidative stress [32].

In the case of amino acids, transporter systems in cartilage chondrocytes have not yet been identified, but glycine, proline, glutamine and glutamate transporters in chondrocytes have recently been investigated [53,54]. Amino acids are expected not only to control chondrocyte gene expression [55] but also to synthesize collagen by chondrocytes [56]. Therefore, amino acids in NMS were selected to provide substrates for fibril forming collagen and PG, based on their prevalence in the triple-helical structure of collagen, and depending on their specific biochemical and physiologic characteristics [21,25]. Some amino acids' abilities to maintain cartilage integrity have already been revealed. For example, hydroxyproline stabilizes the collagen fibres to hold water [33] and glutamate prevents cartilage calcification [37,38]. Glutamine protects articular chondrocytes from heat stress and nitric oxide induced apoptosis [28], it regulates collagen gene expression in cultured human fibroblasts [36], and it also increases collagen gene transcription [31]. Arginine and lysine increase insulin-like growth factor-1 production and collagen synthesis [29]. Lysine also slows the loss of collagen and PG from disrupted articular cartilage surfaces [32]. Methionine stimulates synthesis and deposits of PG in articular cartilage [30,34], and cysteine activates a signalling pathway in articular chondrocytes [27] and protects chondrocytes and cartilage from oxidative damage and degenerative processes such as OA [35]. Therefore, sufficient nutrients from the metabolically active synovium reach the chondrocytes, presumably by diffusion through the cartilage matrix via the synovial fluid and various transporter systems. Finally, all of the components of NMS cooperate with each other in promoting chondrocyte activities to regenerate a collagen and PG network, and in preventing degenerative changes in articular cartilage. This is the great benefit of intra-articular injection of NMS, and one that existing OA treatments can not provide.

Existing OA treatments, such as intra-articular injections of either glucose or dextrose solution (5–25%) alone, are expected to yield osmotic changes and production of precursors for extracellular matrix macromolecules in the articular cartilage [24]. Hypertonic solution is known to generate a better therapeutic effect, but it causes more discomfort from an inflammatory reaction [19,57,58]. The osmotic change in the knee joint cavity induced by 10% hypertonic dextrose, which we used in this study, is as follows: [296 (synovial fluid) + 505 (10% dextrose)]/2 = 400.5 mOsm. This osmolarity of 400 mOsm is of excellent therapeutic value, because it exerts a strong influence on proliferation of cells such as chondrocytes, osteocytes and fibroblasts [19]. It also influences protein synthesis and amino acid (proline) transport without any cellular toxicity [59], and produces less pain than 20% dextrose solution does [41]. Oral administration of glucosamine or chondroitin sulphate, which is a component of PG, plays a role as a symptomatic slow-acting drug in degenerative OA, but its effect is slow, small, or temporary [5,60]. Intra-articular injection of hyaluronic acid plays a role in cartilage as a lubricant that lessens the frictional resistance of the cartilage [15], but it only generates temporary or placebo effects [61]. Repeated injections of hyaluronic acid may deteriorate chondrocytes' PG biosynthetic ability [62], because hyaluronic acid is not a substrate for PG but a terminal material. Chondrocyte proliferation therapies, such as arthroscopic abrasion of the articular surface [7], osteotomy [63], transplantation of chondrocytes [8] or soft-tissue grafts [10,64,65], injections of growth factors [9,66] and autologous blood [67], are also administered into the articular cartilage to stimulate proliferation of chondrocytes and repair cartilage matrix. However, these procedures are too expensive for general use, or they require long-term follow up because of the potential risk for cancer [68] or haemorrhagic arthritis [67].

Generally, cartilage in mature rabbits does not readily regenerate [69], and so histological changes after ACLT in rabbit knees, including cartilage hypertrophy, reduced cell density and matrix alterations preceding cartilage fibrillation, lead to progressive degeneration of cartilage [70]. With ageing, the nutritional supply of cartilage diminishes because of degenerative changes of the joint cavity and decreased metabolism. However, if joint cavities were supplied with sufficient nutrients, they might recover from the nutritional deficiency caused by ageing, and OA progression might be inhibited. In this regard, intra-articular injection of NMS has the potential to induce chondrocytes to synthesize a collagen and PG network, which in turn maintains the cartilage matrix and protects against OA progression in the mature rabbit model, whereas NS injection has no such effect.

In summary, 0.5 ml of NMS or NS was intra-articularly administered into the knee joint cavity of mature rabbits for 13 consecutive weeks starting on week 6 after ACLT at 2-week or 3-week intervals, when arthritic changes had begun. It was found that only NMS injection significantly restored the extracellular matrix and inhibited the progression of OA-like changes in articular cartilage that had undergone ACLT. We suggest further comparative studies with other existing OA treatments, because in this study we only examined the effects of NMS on OA progression in comparison with a control (NS) treatment.

## Conclusion

This study is the first trial to administer intra-articularly injectable material, not in the form of a macromolecular compound but in the form of a mixture of elementary nutrients, into the osteoarthritic articular cartilage. Each composition of the mixture, NMS, is likely to promote upregulated energy production in chondrocytes and extracellular matrix metabolism in articular cartilage, and to exert antioxidative effects in ageing chondrocytes. Based on the results of this study, NMS injection may be applied to osteoarthritic articular cartilage of adult animals as a very simple and effective treatment without significant adverse effects.

## Abbreviations

ACLT = anterior cruciate ligament transection; H&E = haematoxylin and eosin; NMS = nutritive mixture solution; Normal = normal articular cartilage; NS = normal saline; NSAIDs = nonsteroidal anti-inflammatory drugs; OA = osteoarthritis; PG = proteoglycan; SEM = scanning electron microscopy.

## Competing interests

SWL and IHL have a patent on NMS in South Korea.

## Authors' contributions

YSP participated in the design of the study, performed animal surgery, analyzed and interpreted the data, and drafted and revised the manuscript. SWL and IHL conceived the study, participated in its design and coordination, performed animal surgery, and helped to draft the manuscript. TJL participated in the study design, performed histopathological examinations, interpreted the data, and reviewed the manuscript. JSH and JSK participated in the study design, animal handling and care, and reviewed the manuscript. All authors read and approved the final manuscript.

## Figures and Tables

**Table 1:** Compositions of the NMS

Compositions	Contents of 100 ml solution	Contents of 0.5 ml NMS
Dextrose solution^a^	20.0 g (20%)	0.25 ml
Amino acids solution^b^	20.0 g (20%)	0.20 ml
Glycine	4.0 g	0.008 g
Proline	4.0 g	0.008 g
Hydroxyproline	2.0 g	0.004 g
Glutamate	2.0 g	0.004 g
Alanine	1.0 g	0.002 g
Aspartate	1.0 g	0.002 g
Serine	1.0 g	0.002 g
Glutamine	0.5 g	0.001 g
Arginine	1.0 g	0.002 g
Lysine	1.0 g	0.002 g
Methionine	1.5 g	0.003 g
Cysteine	1.0 g	0.002 g
Ascorbic acid solution^b^	5.0 g (5%)	0.05 ml

^a^Dextrose solution (20%) was purchased from Daehan Pharmaceutical Co. Ltd. ^b^Amino acids and ascorbic acid were purchased from Sigma Co. Ltd., and they were dissolved in sterilized distilled water. NMS, nutritive mixture solution.

**Table 2:** Histopathologic evaluation of H&E stained cartilage by the Mankin grading method

Items	Normal (*n *= 7)	NMS (*n *= 7)	NS (*n *= 11)	P*
Medial tibial plateau				
Structure	0.57 (0.30)^a^	2.14 (0.46)^a^	5.27 (0.60)^b^	0.000
Cell in tangential zone	0.29 (0.18)^a^	0.71 (0.18)^a^	1.50 (0.15)^b^	0.000
Cell in transitional and radial zone	1.00 (0.69)^a^	2.57 (0.43)^a^	6.05 (0.75)^b^	0.000
Pannus formation	0.00 (0.00)^a^	0.29 (0.18)^a^	1.18 (0.30)^b^	0.005
Sum of scores	1.86 (1.12)^a^	5.71 (0.84)^b^	14.00 (0.75)^c^	0.000
Medial femoral condyle				
Structure	0.57 (0.30)^a^	1.29 (0.47)^ab^	2.50 (0.54)^b^	0.031
Cell in tangential zone	0.29 (0.18)^a^	0.43 (0.20)^ab^	0.95 (0.11)^b^	0.010
Cell in transitional and radial zone	1.00 (0.69)^a^	2.29 (0.68)^a^	5.36 (0.78)^b^	0.001
Pannus formation	0.00 (0.00)	0.43 (0.20)	0.73 (0.24)	0.062
Sum of scores	1.86 (1.12)^a^	4.43 (1.17)^a^	9.55 (1.30)^b^	0.001

Data represent the means (standard error) for each group. As the severity of cartilage lesions increases, so does the score obtained using the Mankin grading method. The Normal group includes normal articular cartilage group that did not undergo any surgery or treatment. The nutritive mixture solution (NMS) group received NMS after surgery. The normal saline (NS) group received NS after surgery. *Differences among the three groups were considered to be statistically significant when the *P *value was under 0.05 by one-way analysis of variance and Tukey HSD test, or Kruskal-Wallis test, depending on normality of data. ^a,b,c^Data with different letters in the three groups are statistically significantly different.
